# Obituary

**Published:** 1846-07

**Authors:** S. G. Ellis

**Affiliations:** Lodi


OBITUARY.—Died, June 21, 1846, in the town of Collins, Erie county, N. Y., ISRAEL CONGDON, in the 46th year of his age. Dr. C. graduated at Fairfield, Herkimer Co., N. Y., in 1828; soon after which he located in Collins, where, by his medical skill, he soon won the confidence of the public, and by his sterling integrity secured their unaffected esteem and friendship. Dr. C.'s sickness was severe and protracted, having been confined to his couch for more than two years. The esteem in which Dr. C. was held was manifest in the vast concourse of people who attended his funeral. In the death of Dr. C. the profession has lost a member who was an ornament to it, and the community a much esteemed physician. an upright citizen, a kind neighbor. The sick and the afflicted have emphatically lost a sympathising friend.

LODI, June 26, 1846. S. G. ELLIS, M. D.

